# The efficacy of fecal microbiota transplantation for patients with chronic pouchitis: A case series

**DOI:** 10.1002/ccr3.2096

**Published:** 2019-03-12

**Authors:** Atsushi Nishida, Hirotsugu Imaeda, Osamu Inatomi, Shigeki Bamba, Mitsushige Sugimoto, Akira Andoh

**Affiliations:** ^1^ Department of Medicine Shiga University of Medical Science Otsu Japan; ^2^ Division of Clinical Nutrition Shiga University of Medical Science Otsu Japan; ^3^ Division of Digestive Endoscopy Shiga University of Medical Science Otsu Japan

**Keywords:** fecal microbiota transplantation, pouchitis, ulcerative colitis

## Abstract

Pouchitis is one of the most common complications that develop after restorative proctocolectomy with ileal pouch‐anal anastomosis for ulcerative colitis. Single fecal microbiota transplantation (FMT) by colonoscopy was performed safely on three patients with pouchitis. However, the efficacy of FMT on pouchitis was limited.

## 1 INTRODUCTION

Ulcerative colitis (UC) is a diffuse nonspecific chronic inflammation of the colon characterized by erosion or ulcer in the mucosa.^1^, ^2^ It has been reported that about 30% of patients with UC require surgical treatment at some time in their lives.^3^, ^4^ Surgical indications for UC include colonic perforation, life‐threatening gastrointestinal hemorrhage, toxic‐megacolon, resistant to medical treatment, dysplasia, and colon cancer.^5^, ^6^ Most patients undergo a restorative proctocolectomy (RPC) with ileal pouch‐anal anastomosis (IPAA). RPC with IPAA removes the entire colon and rectum while preserving the anal sphincter and, hence, normal bowel function and fecal continence. The pouch serves as an internal pelvic reservoir for intestinal contents.^7^ Therefore, RPC with IPAA has made surgical management a more attractive option than a total proctocolectomy with a permanent end ileostomy. However, there are some patients who develop inflammation in the ileal pouch, a condition called pouchitis.

Pouchitis, which is a term referring to an inflammatory condition of the ileal pouch reservoir, is the most frequently observed long‐term complication occurring in patients with IPAA. Pouchitis occurs almost exclusively in patients with UC and rarely in patients with familial adenomatous polyposis.^8^ Among patients with UC, the risk of pouchitis is increased in patients with extensive colitis.^8^ The prevalence of pouchitis ranges from 23% to 46%, with an annual incidence up to 40%, and about 5% of these patients develop chronic pouchitis requiring immunomodulators, biologics, or a resection of the pouch.^9^, ^10^

Although the cause of pouchitis remains unknown, it is hypothesized to result from an abnormal immune response to altered gut microbiota in genetically susceptible hosts. Previous reports support this assumption. First, it has been reported that the administration of antibiotics is effective for pouchitis.^11^, ^12^ Second, it has also been reported that the administration of a probiotic, VSL # 3, is effective in the prevention of pouchitis.^11^, ^13^ In addition, it has been reported that several genes associated with the innate immune response and bacterial recognition mechanisms such as NOD2/CARD15 gene,^14^ toll‐like receptor gene,^15^, ^16^ and IL‐1 receptor agonist gene^17^ increase the risk of pouchitis.

Fecal microbiota transplantation (FMT) is a novel therapeutic procedure that aims to restore the composition of gut microbiota by transferring normal intestinal flora from a healthy donor to a patient. FMT has been recently applied to various kinds of human diseases associated with dysbiosis such as*Clostridium difficile* infection,^18^, ^19^, ^20^ inflammatory bowel disease,^21^, ^22^, ^23^ irritable bowel syndrome,^24^ type 2 diabetes,^25^ and autism spectrum disorders.^26^ In particular, it has been reported that FMT exhibited high efficacy in the treatment of recurrent *Clostridium difficile* infection that was otherwise resistant to medical treatment.^19^ To date, there are a few reports available about the efficacy of FMT for pouchitis. Moreover, there is no study about the efficacy of FMT on Japanese patients with pouchitis. Therefore, the efficacy of FMT for pouchitis remains unknown. In this study, we performed FMT on three patients with pouchitis after RPC with PIAA for UC and examined the efficacy and safety.

## 2 MATERIALS AND METHODS

### 2.1 Ethics

The protocol was approved by the Institutional Review Board of Shiga University of Medical Science (Permission No. 26‐184). All participants provided written informed consent. This study was registered with the University Hospital Medical Information Network Center (UMIN000016900).

### 2.2 Study design and patients

This was an open‐label case series performed at Shiga University of Medical Science from January 2015 to June 2016. Eligible patients were older than 15 years with pouchitis defined as a current pouchitis disease activity index (PDAI) ≥7. Exclusion criteria included intestinal cytomegalovirus infection, pregnancy, current serious disease, and participation in other clinical studies.

### 2.3 FMT donors

Healthy relatives within the second‐degree relationship (aged 18 years or older) were screened by stool and serological tests as previously reported.^23^

### 2.4 FMT procedure

We performed FMT as previously reported.^23^ Briefly, fresh feces from donors (approximately 150‐200 g) obtained within 4 hours before FMT were dissolved in 500 mL of sterile physiological saline (350‐500 mL) and filtered through sterile gauze to remove crude components. Fecal material was administered to the patients via colonoscopy following standard bowel preparation with polyethylene glycol solution (Niflec®, EA Pharma, Tokyo, Japan). Endoscopic delivery of fecal material was performed proximal to the pouch.

### 2.5 Clinical outcomes

The primary endpoint was a clinical response at 8 weeks after FMT. A clinical response was defined as a reduction in total PDAI score by 3 ≥ points. A clinical remission was defined as a reduction in total PDAI score by 3 ≥ points and total PDAI score < 7. PDAI was checked every 2 weeks up to 8 weeks. The fecal samples were obtained every 4 weeks up to 8 weeks.

### 2.6 DNA extraction

Bacterial DNA was isolated as described previously.^23^ Briefly, a fecal sample (0.5 g) was suspended in 5 mL of tris (hydroxymethyl) aminomethane‐EDTA buffer (pH 7.5) and centrifuged. This washing step was performed four times. The sample was then resuspended in 5 mL of the same buffer containing lysozyme (5 mg/mL; Sigma, St Louis, MO), *N*‐acetylmuramidase (0.5 mg/mL; Sigma), and achromopeptidase (0.5 mg/mL; Sigma).

### 2.7 Polymerase chain reaction amplification and terminal restriction fragment length polymorphism analysis

Polymerase chain reaction (PCR) and terminal restriction fragment length polymorphism (T‐RFLP) analyses were performed according to the method described previously.^23^

## 3 RESULTS

### 3.1 Case histories and outcomes

Three patients with pouchitis were enrolled in this study, and FMT was performed on all three patients. The basic characteristics of the patients are shown in Table 1.

**Table 1:** Baseline characteristics of patients with pouchitis who underwent fecal microbiota transplantation

Patients	Patient 1	Patient 2	Patient 3
Age (y)	24	45	52
Sex	Male	Male	Female
Disease type	Total colitis	Total colitis	Total colitis
Previous treatment	Anti‐TNF‐α antibody	VSL#3	Ciproxan, metronidazole
Pouch type	J‐pouch	J‐pouch	J‐pouch
Pouch age (mo)	54	108	96
Duration of pouchitis (mo)	48	84	60
Hemoglobin (g/dL)	13.1	12.2	13.8
ESR (mm/h)	8.0	49.0	14.0
CRP(mg/dL)	1.14	0.71	0.28
Albumin (g/dL)	4.3	3.3	4.1
PDAI	9	15	12

CRP, C‐reactive protein; ESR, erythrocyte sedimentation rate; PDAI, pouchitis disease activity index.

Patient 1 was a 24‐year‐old Japanese man. He was suffering from UC and had been diagnosed as fulminant type at the age of 20. RPC with IPAA was performed. He developed pouchitis 6 months after surgery and was treated with antibiotics. However, he later developed chronic antibiotics‐resistant pouchitis with a relapsing and remitting pattern. Constantly, treatment with anti‐TNF‐α antibody (adalimumab) was started but the condition did not improve. The decision was then made to proceed with FMT for pouchitis, and this was performed following the screening of donors. The PDAI score before FMT was 9 points, and this decreased to 7 points at 8 weeks after transplantation. Neither a clinical remission nor a clinical response due to FMT was achieved. No adverse events were observed either after FMT or during the follow‐up period (Table 2).

**Table 2:** Outcome of fecal microbiota transplantation and adverse events

	PDAI before FMT	PDAI after FMT	Clinical remission	Clinical response	Adverse event
Patient 1	9	7	No	No	No
Patient 2	15	14	No	No	No
Patient 3	12	7	No	Yes	No

FMT, fecal microbiota transplantation; PDAI, pouchitis disease activity index.

Patient 2 was a 45‐year‐old Japanese man who had suffered from UC since the age of 30 years. He later developed severe UC that was resistant to medical treatment, and RPC with IPAA was performed. He developed pouchitis 48 months after surgery and was treated with antibiotics but his condition subsequently progressed to chronic antibiotic‐resistant pouchitis. Administration of a probiotic (VSL # 3) was then started but no improvement was observed. The decision was then made to proceed with FMT for chronic pouchitis. The PDAI score before FMT was 15 points, and this decreased to 14 points at 8 weeks after transplantation. Neither a clinical remission nor a clinical response due to FMT was achieved. No adverse events were observed either after FMT or during the follow‐up period (Table 2).

Patient 3 was a 52‐year‐old Japanese woman who had suffered from UC since the age of 36 years. She later developed severe UC resistant to medical treatment at the age of 46. She underwent RPC with PIAA but developed pouchitis 36 months after surgery. A course of antibiotics was started, and the symptoms of pouchitis initially improved before relapse with frequent recurrence. She later developed chronic antibiotic‐resistant pouchitits. The PDAI score before FMT was 12 points, and this decreased to 7 points at 8 weeks after transplantation. A clinical remission was not achieved but there was a clinical response. No adverse events were observed either after transplantation or during the follow‐up period (Table 2).

In the three patients who underwent FMT for pouchitis after RPC with IPAA, a clinical remission was not achieved in any of the cases, but a clinical response was achieved in one case. In addition, no adverse events were observed either after FMT or during the follow‐up period.

### 3.2 Fecal bacterial analysis in donors and patients

**Figure 1:**
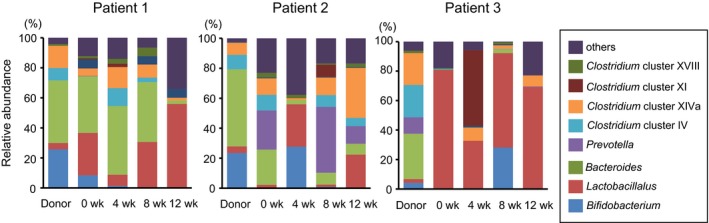
Composition of the gut microbiota in donors and patients before and after fecal microbiota transplantation (FMT). The gut microbiota of donors and patients before and after FMT was analyzed by terminal restriction fragment length polymorphism. The value indicates the percentage of the predicted bacteria

We investigated bacterial composition in feces using TRFL‐P. Fecal samples from donors and patients before FMT, and 4 and 8 weeks after FMT were examined (Figure 1).

**Figure 2:**
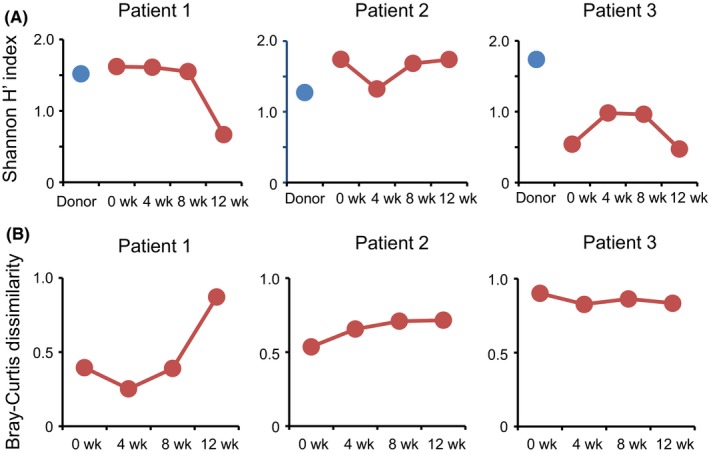
Bacterial diversity and the similarity of microbial composition. A, Shannon diversity index of gut microbiota in patients before and after fecal microbiota transplantation (FMT) (weeks 4, 8, and 12) and their respective donor. B, The similarity of microbial composition between patient's feces before and after FMT (weeks 4, 8, and 12) and their respective donor sample

In Patient 1, who did not achieve a clinical response at 8 weeks after FMT, the Shannon diversity index for donor feces did not change at 4 and 8 weeks after FMT (Figure 2A). The similarity of microbial composition between the patient's sample after FMT and the donor's sample was examined using the Bray‐Curtis dissimilarity index. There was no remarkable change in Bray‐Curtis dissimilarity index at 4 and 8 weeks after FMT (Figure 2B).

In Patient 2, who did not achieve a clinical response at 8 weeks after FMT, the Shannon diversity index of the patient did not change at 4 and 8 weeks after FMT. The similarity of microbial composition between the patient's sample after FMT and the donor's sample was examined using the Bray‐Curtis dissimilarity index. There was no remarkable change in Bray‐Curtis dissimilarity index at 4 and 8 after FMT (Figure 2B).

In Patient 3, who achieved a clinical response at 8 weeks after FMT, the Shannon diversity index for donor's feces did not change remarkably at 4 and 8 weeks after FMT. Furthermore, there was no change at 4 and 8 weeks after FMT in the Bray‐Curtis dissimilarity index (Figure 2B).

Collectively, these results indicated that a single FMT using colonoscopy did not show sufficient effect to change the composition and the diversity of gut microbiota of the patients.

## 4 DISCUSSION

In this study, we examined the efficacy and safety of a single FMT using colonoscopy for Japanese patients with pouchitis after RPC with IPAA. A clinical remission was not achieved in any of the patients, while a clinical response was achieved in one patient. Moreover, no adverse events were observed either after FMT or during the follow‐up period. Thus, our results indicate that single FMT for patients with pouchitis after RPC with IPAA could be performed safely, but its clinical effects were limited.

Recent studies have shown that gut microbiota plays an important role in the pathogenesis of various kinds of human diseases.^27^, ^28^, ^29^ It has been reported that the alteration of gut microbiota was observed in patients with pouchitis. A recent study has reported an increase in *Clostridium* and *Fusobacterium*, and a decrease in *Lactobacillus* and *Streptococcus* in the mucosa‐associated gut microbiota of patients with pouchitis as compared to a noninflamed pouch.^30^ Other reports have showed a decrease in the diversity of gut microbiota and a decrease in the abundance of *Faecalibacterium*, *Eubacterium*, and *Roseburia* in patients with pouchitis as compared to a noninflamed pouch.^31^ Thus, FMT may become a therapeutic option for pouchitis after RPC with IPAA for UC to restore the alteration of gut microbiota.

To the best of our knowledge, there are only two reports on the efficacy of FMT on multiple cases of pouchitis after surgery for UC.^32^, ^33^ Stallmach et al reported that FMT was performed on five patients with pouchitis after RPC with IPAA, and a clinical remission was achieved in 3 out of the 5 patients. They suggested that FMT was effective for pouchitis after RPC with IPAA.^33^ Landy et al reported that FMT was performed on eight patients with pouchitis after RPC, and a clinical response was achieved in 2 out of the 8 patients. They concluded that the efficacy of FMT for pouchitis after RPC was limited.^32^ In our study, FMT was performed on three patients with pouchitis after RPC with IPAA, and a clinical response was achieved in 1 out of the 3 patients. These results suggest that the efficacy of FMT for pouchitis after RPC with IPAA is limited. Therefore, some improvements are required in the method of FMT to improve the efficacy of FMT for pouchitis after RPC for UC.

Since the frequency or the route of administration of FMT was different in the previous studies, there is a possibility that these factors affect the efficacy of FMT Stallmach et al^33^ administered donor feces twice to the jejunum using endoscopy. On the other hand, Landy et al^32^ performed a single administration of donor feces to the jejunum using a nasogastric tube. We performed a single administration of donor feces to the ileum using colonoscopy. Collectively, these findings suggest that further investigation on the frequency or the route of the administration of FMT is required to improve the effectiveness for pouchitis. Furthermore, the selection of donors was also different in the previous studies. Stallmach et al^33^ selected healthy unrelated volunteers as donors, and Landy et al^32^ selected relatives, partners, or unrelated volunteers as donors. We selected donors from healthy relatives within the second degree of relationship. These results provide no conclusive evidence as to who is suitable as a donor. Further investigation on donor selection is therefore merited.

In the previous studies, the gut microbiota of patients with a clinical response successfully changed to a composition of gut microbiota similar to that of donors.^32^, ^33^ Landy et al^32^ demonstrated that the similarity analyzed by Bray‐Curtis dissimilarity index indicated a shift in the stool microbiota of responders toward a composition with greater similarity to donors stool at 4 weeks after FMT. Stallmach et al suggested that in the responders, the stool microbial composition successfully changed to a structure similar to that of donors at about 4 weeks after FMT, whereas the nonresponders showed a unique pattern distinct from the microbiome of the donor.^33^ In our study, there was no remarkable shift in the composition of gut microbiota of patients to that of donors by FMT even in the patient with a clinical response. These results suggest that it is necessary to improve the method of FMT to change the microbial composition of patients to be similar to those of donors.

In summary, this is the first study to evaluate the efficacy and safety of FMT in Japanese UC patients with pouchitis after RPC with IPAA. Although FMT by colonoscopy was performed safely in all patients, we could not confirm sufficient efficacy of FMT in patients with pouchitis. In the future, FMT may be a promising option for treating pouchitis otherwise resistant to medical treatment. Therefore, the accumulation of data on FMT‐treated patients with pouchitis and randomized controlled trials for the efficacy of FMT for pouchitis are necessary. Further investigation of the frequency and administration route of FMT, and the donor selection are required. These issues should be resolved in order to improve the efficacy of FMT for patients with pouchitis.

## CONFLICT OF INTEREST

The authors declare that they have no competing interests.

## AUTHOR CONTRIBUTION

AN: analyzed the data and drafted the manuscript. HI, OI, SB, MS, and AA: contributed to the drafting.
